# Neutrophil to Lymphocyte Ratio in Lung Cancer: Implications for Depressive Symptoms and Survival

**DOI:** 10.31487/j.cor.2020.06.12

**Published:** 2020-06-19

**Authors:** Daniel C. McFarland

**Affiliations:** Department of Psychiatry and Behavioral Sciences, Memorial Sloan Kettering Cancer Center, New York, USA

**Keywords:** Neutrophil to lymphocyte ratio, lung cancer, depression, survival, depressive symptoms, anxiety, prognostic marker

## Abstract

**Background:**

Depression very commonly appears in the presence of lung cancer. Multiple contexts have shown that depression is associated with inflammation. The Neutrophil to Lymphocyte Ratio (NLR) provides an easy to interpret the measure of both inflammation and immunity, but its use as an inflammatory biomarker has not been evaluated in patients with lung cancer. We hypothesize that NLR will be elevated in depressed patients with lung cancer and that both elevated NRL and depression will have prognostic implications.

**Methods:**

Patients (n=109) were assessed for depression and anxiety using the Hospital .Anxiety and Depression Scale (HADS) and for distress using the Distress Thermometer. NLR was derived from a complete blood count obtained on the day of the cross-sectional survey. Data were dichotomized (NLR ≥5 and HADS-D ≥8) and analysed for survival estimations using Kaplan-Meier plots.

**Results:**

NLR was found to be significantly correlated with depression (r=.21, p=.03) and was associated with depression while controlling for age, sex, and marital status (β=.21, p=.004). NLR trended toward correlation with anxiety (r=.19, p=.07). Elevated NLR (≥5) predicted for worse survival (chi square= 10.08, p=.001), which was similarly seen when combined with meeting depression criteria (chi square = 16.00,p<.001).

**Conclusion:**

NLR provides a reasonable assessment of lung cancer related inflammation with survival implications that may indicate the presence of depression. These results warrant further research.

## Introduction

Depression commonly co-occurs with lung cancer [1]. Depression is associated with inflammation in patients with cancer and other chronic medical illness as well as medically healthy patients [2, 3]. Specifically, the association between lung cancer and depression has been shown using various inflammatory markers [4]. Understanding the characteristics of this descriptive association has important implications for cancer-related quality of life, overlapping symptoms also related to inflammation (e.g., fatigue, sleep disturbances, pain), and the potential for directed treatment applications addressing levels of inflammation or the effects of inflammation that appear to cause depressive symptoms (i.e., depletion of the neurotransmitter dopamine) [5–11]. Historically, depression associated with cancer, co-morbid medical illness, or inflammation conferred depression treatment resistance [12, 13].

Exciting initiatives now incorporate the presence of chronic inflammation as a gateway to address the underlying biological underpinnings of depression, holding promise for rationally directed depression treatments in patients with medical illnesses such as cancer [14].

However, inflammation is a multifaceted process that can be acute or chronic with an innate predisposition and variable clinical sources to induce inflammation. The Neutrophil to Lymphocyte Ratio (NLR) provides a broadly nonspecific measure of both inflammation and immunity [15]. The NLR is a ratio of absolute neutrophil count over absolute lymphocyte count and therefore rises when either inflammation increases or lymphocytes get depleted through immune activation. Depression appears to affect both inflammation and immunity, mostly through T cell dysregulation [16]. Commonly, general chronic inflammation is measured by either an array of pro- and anti-inflammatory cytokines such as interleukin-6 (IL-6) or tumor necrosis factor α (TNF-α) of is measured indirectly by acute phases reactants such as C-reactive protein (CRP), which become elevated in response to pro-inflammatory cytokines, mostly IL-6.^2^ There is less robust literature representing the association between depression and T cell (lymphocyte) dysfunction [17].

The NLR has been evaluated extensively as a prognostic marker across various cancer types. Several studies have shown that it is predictive of worse prognosis at the time of surgery or while undergoing treatment, for example [18]. It has also been used as a prognostic marker in critical care settings such as sepsis [19]. As opposed to examining pro-inflammatory cytokines, the measurement of NLR is easily derived from a complete blood count that can be run in any lab and does not have the same diurnal variation and short half-life. The acute phase reactant CRP is also fairly easy to interpret, but these tests are not routinely done in patients with cancer. It is important to remember that while many different processes can cause gain in the elevation of inflammation, the end result is depression, no matter what the source of inflammation. However, it is important to remember all the factors that may contribute to larger NLR or greater inflammation such as smoking, obesity, advancing cancer, medications, and sex.

A meta-analysis found that NLR was higher in non-medically ill patients (non-cancer patients) with depression and bipolar disorder than healthy controls similar to other markers of inflammation [20]. However, in the cancer setting, only one study has evaluated the relationship between NLR and depression, which was among 53 patients with gastric cancer prior to surgery. The investigators found that NLR was indeed associated with depressive symptoms [21]. At the same time, more studies are needed given the complexities of using NLR in the cancer setting where patients are receiving immunosuppressive chemotherapy, immunotherapy and other drugs that will influence NLR.

This study sought to evaluate the relationship between NLR and depression in patients with metastatic lung cancer who are receiving active treatments. The importance of detecting depression early in the course of lung cancer is imperative to improve patient outcomes. Despite best efforts at distress and depression screening, depression continues to be undertreated [22]. Lung cancer has one of the highest rates of co-morbid depression among all cancer types (16-29%) [23, 24]. The identification of easy to use biomarkers such as NLR may facilitate the identification and treatment of depression while contributing to understanding the underlying etiology of depression in cancer settings.

## Methods and Materials

The Memorial Sloan Kettering Cancer Center Institutional Review Board (IRB) approved this study in May 2018. Routine blood work and depression screening questionnaires were collected as a standard of care practice from May 2017 to November 2017. These results are part of a larger study that was reported previously [25].

### I Participants

Men and women with histologically confirmed stage IV lung cancer who were undergoing active treatment, spoke English, and had a performance status of ECOG 2 or greater were included. Participants with other cancers or not undergoing treatment for stage IV lung cancer were excluded. Participants had to be on active treatment for at least one month and had to be more than one month from receiving the diagnosis of lung cancer to be included.

### II Procedure

Patients were asked to complete a one-time survey by a treating staff member (e.g., nurse practitioner, medical oncologist). They filled out the questionnaire containing standardized survey questions, and CRP laboratory values were obtained the same day that the questionnaires were completed. Available psychological services were provided in the survey, and patients were asked to raise any concerns with clinic staff and, in particular, to tell a staff member if they felt significantly depressed or had suicidal ideation. The surveys were collected as a routine standard of care screening as per distress screening guidelines.

### III Measures

#### i Patient Demographic and Medical Characteristics

Participant demographic information was obtained from the medical record and included age, race/ethnicity, sex, marital status, body mass index (BMI), length of time since diagnosis, type of treatment (e.g., chemotherapy, immunotherapy, targeted therapy), line of treatment (i.e., 1^st^, 2^nd^, 3^rd^ or beyond), and whether they were taking an antidepressant medication.

#### ii Biological Characteristics (NLR)

Neutrophil-to-Lymphocyte Ratio (NLR): NLR was obtained from the complete blood count. The ratio was calculated by dividing the absolute neutrophil count by the absolute lymphocyte count. The value was obtained in a Clinical Laboratory Improvement Amendments (CLIA) certified lab [26]. Inter- and intra-assay coefficient of variation is reliably less than 5%. NLR cut-off of 5 has been used as a dichotomous predictive marker of survival [27].

#### iii Depression and Anxiety

Depression severity was measured by the Hospital Anxiety and Depression scale (HADS), which has been validated in the lung cancer setting [28, 29]. The HADS is a 14-item symptom rating scale that was developed to identify clinically significant cases of anxiety and depressive disorders among medically ill patients.^28^ Unlike most symptom rating scales, physical symptoms are excluded from the HADS due to the potential confounding effects of illness on symptoms such as sleep, appetite disturbance, and fatigue. The HADS is divided into an anxiety subscale (HADS-A) and a depression subscale (HADS-D). Responses are rated 0 to 3 points such that total scores on the HADS-A and HADS-D may range from 0 to 21 points. A cut-off of 8 on the HADS-D subscale is most commonly used to identify clinically significant depression, with an average sensitivity and specificity of 0.80 [28, 30]. Because research on inflammation has focused primarily on depression, not anxiety, only HADS-D data were analysed in this study.

#### iv Distress

Tlie DT&PL is a one-item measurement that ranges from 0 to 10 and is recommended by the National Comprehensive Cancer Network to screen for psychological distress in order to meet their distress screening guidelines and the Commission on Cancer distress screening mandate [31]. A cut-off of ≥4 has been accepted by the NCCN to indicate clinically meaningful distress [32]. The DT has shown strong discriminatory power relative to depression (derived from the PHQ-9) with an area under the curve of 0.87. Similar operating characteristics were seen with variations in age, education, marital status and stage of the disease.

### IV Statistical Analysis

Statistical procedures were performed using the SPSS version 24 software (SPSS, Chicago, IL 2013), and all statistical tests were two-tailed with a 5% significance level. The primary outcome of this study was the severity of depressive symptoms, and in particular, its association with NLR in patients with metastatic lung cancer. Because NLR data are not normally distributed, NLR values were log-transformed prior to data analysis; however, untransformed values are also reported for ease of interpretation. Univariate associations between patient demographic and medical characteristics with depression (HADS-D) and NLR were assessed with Spearman correlation coefficients (rank-order correlations for age, BMI, time since diagnosis), independent t-tests (dichotomous variables of race/ethnicity, marital status, antidepressant use), and ANOVAs (categorical variables of type of treatment, disease status). NLR values were dichotomized at 5 to create high and low NLR values. These were used in the Kaplan Meier plot survival analysis.

## Results

The study surveyed 140 potential participants, and 109 returned survey information (77.9% response rate). Table 1 highlights the cohort characteristics. Clinically significant depression was endorsed by 23.9% (HADS-D≥8). Eighteen (16.5%) participants reported that they were taking antidepressant medication, including only seven out of the 26 participants (26.9%) with clinically significant depression. There was no association between antidepressant use and the severity of depressive symptoms. There was no evidence that antidepressant use mediated or moderated the effect of inflammation on depression when included as an interaction term. (*p*=.45).

The average NLR was 7.36 (SD 12.6), with an average absolute neutrophil count of 5.53 (SD 3.4) and an absolute lymphocyte count of 1.28 (SD 3.4). There was no significant variation in NLR for patients on different types of systemic therapy (chemotherapy, immunotherapy, targeted therapies) (p=.60) or for patients with different types of lung cancer (p=.83), or on different lines of treatment (p=.91) (Table 2).

NLR was associated with depression (r=.21, p=.03), trended toward significance with anxiety (r=.19, p=.07) but was not associated with distress. On multivariate analysis, NLR was associated with depression when controlling for age, sex, and marital status (β=.21, p=.004; adjusted R^2^=.16) (Table 3).

**Figure 1:**
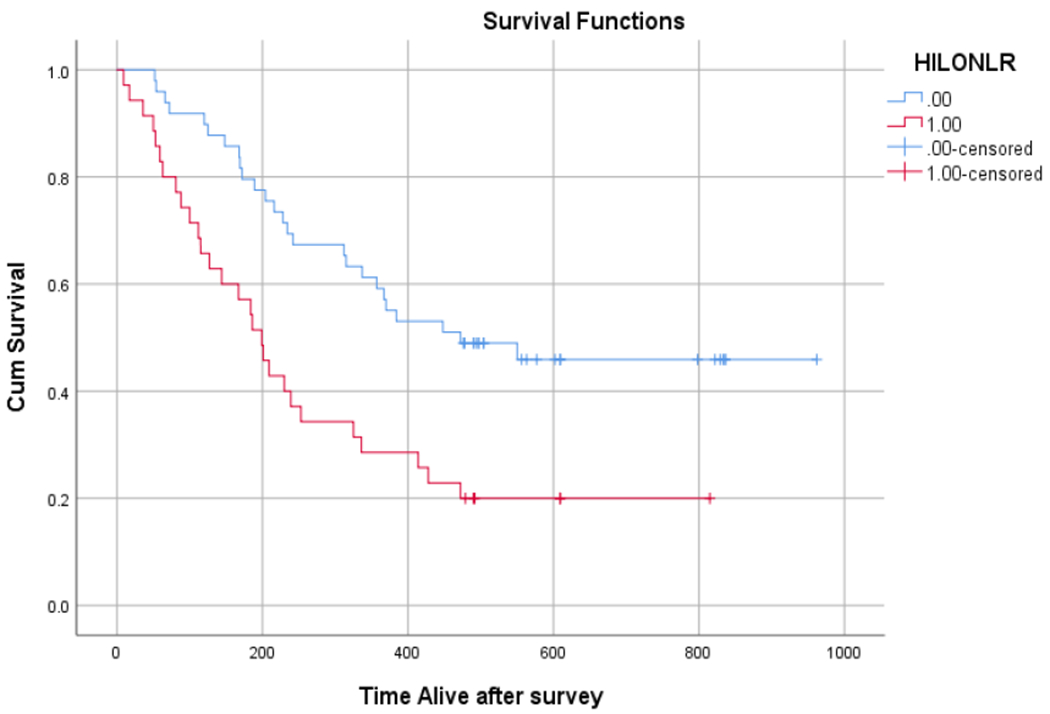
Survival estimation by Kaplan Meier plot using Neutrophil to Lymphocyte Ratio and a cut-off point of ≥5 (red) and <5 (blue).

**Figure 2:**
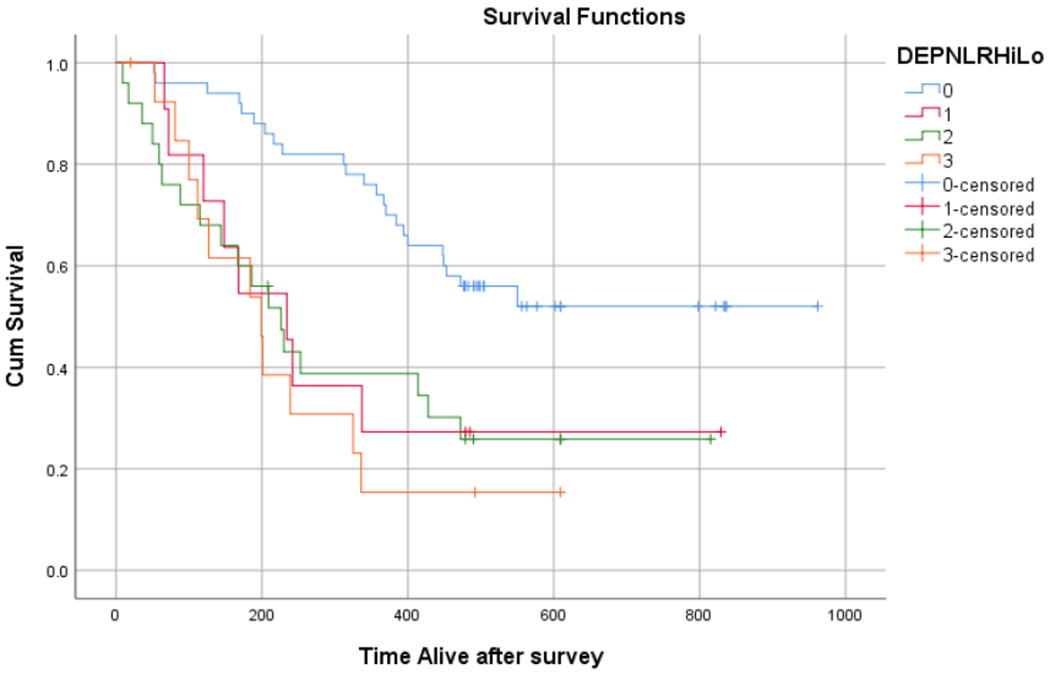
Survival estimation by Kaplan Meier plot using Neutrophil to Lymphocyte Ratio (NLR) cut-off point of 5 (≥5=high and <5=low) and meeting depression criteria (Hospital Anxiety Depression Scale ≥8) to great the following: Blue (0): no depression, low NLR; Red (1): Depression, Low NLR; Green (2): No Depression, High NLR; Orange (3): Depression, High NLR.

On average, patients with NLR ≥5 exhibited worse survival (303 days [95% CI 210 to 395 days]) compared with patients with NLR <5 (577 days [95% CI 473 to 682 days]) (chi square 10.08, p=.001) (Figure 1). When meeting depression criteria (HADS≥8) was factored into NLR cut-off scores, four groups were created with the following survival data: 0) no depression and low NLR (651 days [95% CI 556-747]), 1) depression and low NLR (352 days [95% CI 174 to 530]), 2) no depression and high NLR (344 days [95% CI 223 to 465]), and 3) depression and high NLR (244 days [95% CI 148 to 340]) (Figure 2).

Pair-wise analysis found that survival estimates were statistically superior for group 0 (no depression, low NLR) versus group 1 (depression, low NLR) (chi square 6.66, p=.01), versus group 2 (no depression, high NLR) (chi square 9.52, p=.02), and versus group 3 (depression, high NLR) (chi square 16.00, p<.001) (Figure 2).

## Discussion

NRL is associated with depression in lung cancer both in terms of severity and clinical significance. Both inflammation and depression were elevated and significantly associated in this population of lung cancer patients actively undergoing anticancer treatments. NLR may serve as a prognostic tool as well as a descriptive item for the identification of depression. The use of NLR as both a prognostic and predictive biomarker warrants further research.

This is the first study to evaluate the use NLR and its association with depression in patients with lung cancer. It should be noted that this may extend its use and is particularly meaningful, given its ready availability and ease of laboratory processing. Although previous research displays an association between pro-inflammatory biomarkers and depression in lung cancer, some biomarkers are easier for clinicians to use and provide similarly important information on the state of general inflammation. In addition to NLR, acute phase reactants such as CRP and albumin are similarly available and fairly straightforward to interpret [4, 33].

This research highlights how important it is to have a complete understanding of inflammatory biomarker interpretation. In the past, data on pro-inflammatory cytokines and depression were equivocal, and some of the imprecision could be attributed to short half-lives of pro-inflammatory, diurnal variation, and imprecise laboratory measurement [34] . Indirect measures of inflammation, such as through acute phase reactants and NLR, offer greater clinical applicability even though they are indirect and really show the effects of ongoing inflammation rather than the true components of inflammatory cascades. However, the psychological associations of inflammation are fairly well-established and warrant further research on clinical applicability.

Lung cancer, the most commonly diagnosed cancer, that also confers some of the highest rates of depression has demonstrated variation in terms of inflammation by molecular basis (lung cancer subtype, for example, EGFR mutant) and by the number of cancer-related mutations (tumor mutation burden, TMB). Patients with an actionable mutation such as EGFR have lower inflammation and lower rates of depression [35] . Patients with elevated TMB have elevated inflammation and elevated rates of depression [36].

As expected, NLR provided evidence of its significant prognostic capabilities that also overlap with depression. Previous studies have shown that depression in the setting of lung cancer also carries prognostic implications [37, 38]. However, previous studies have not provided mechanistic explanations to account for the relationship between depression and worse survival. These data suggest that while there is overlap between depression and inflammation accounting for worsened survival, depression without inflammation (low NLR) is also associated with worsened survival, suggesting that the relationship is probably also independent of inflammation as well.

NLR has the potential to serve as a biomarker of depression in the cancer setting and also has prognostic implications. Identification of depression early in the course of lung cancer is challenging, and it is possible that using a readily available biomarker may facilitate the process, and this may also have significant survival benefit [39]. The implication of inflammatory biomarkers in the identification and treatment of depression in cancer settings such as lung cancer remains to be evaluated. These data are hypothesis-generating and warrant further exploration in this area. At the same time, the data are limited by its cross-sectional design with relatively small sample size. The data were also collected at a single institution referral center. Therefore, the results should be replicated but offer further hypothesis-generating information.

In summary, NLR provides a readily available tool for clinicians to ascertain the inflammatory states of patients with cancer, many of whom may also have depressive symptoms. NLR, while extensively studied as a prognostic marker, also adds to the research on inflammation and depression, which is particularly relevant for patients with lung cancer who experience high levels of anxiety and depression.

## Figures and Tables

**Table 1:** Clinical and Demographic Characteristics of the Sample.

	Total (n=109)
	M (SD)
Age (years)	65.9 (9.3)
Body Mass Index	26.1 (5.0)
Time with Disease (months)	15.4 (17.3)
NLR	7.36 (12.6)
Neutrophil count	5.54 (3.4)
Lymphocyte count	1.28 (.7)
Depression Score (HADS-D) (0-21)	4.9 (3.7)
	N (%)
Meets Criteria Screen	
Depression (HADS-D ≥8)	26 (23.9%)
Gender	
Male	41 (37.6%)
Female	68 (62.4%)
Disease Type	
Adenocarcinoma	79 (71.8%)
Squamous cell carcinoma	7 (6.4%)
Small Cell Lung Cancer	18 (16.5%)
Unspecified	5 (4.6%)
Treatment Type	
Chemotherapy	47 (45.2%)
Immunotherapy	35 (33.7%)
Targeted Therapy	22 (21.2%)
Missing	6 (5.5%)
Line of Treatment	
1^st^	56 (53.3%)
2^nd^	34 (32.4%)
3^rd^ or beyond	15 (14.3%)
Missing	5 (4.5%)
Race/Ethnicity	
Black	7 (6.4%)
White	93 (85.2%)
Latino	7 (6.4%)
Asian	2 (1.8%)
Married	
Yes	76 (69.7%)
No	33 (30.3%)
Antidepressant	
Yes	18 (16.5%)
No	91 (83.5%)

HADS-D: Hospital Anxiety and Depression Scale-Depression; NLR: Neutrophil to Lymphocyte Ratio.

**Table 2:** Univariate analyses of demographic, physiologic and treatment characteristics related to albumin.

Neutrophil to Lymphocyte Ratio		
	Univariate Analysis	
Variable	r	p
Age	.03	.77
BMI	−.11	.29
Time with Disease	.16	.11
Distress	.09	.41
Anxiety	.19	.07
Depression	.21	.03
	**F**	**P**
Line of Treatment	.51	.91
1^st^		
2^nd^		
3^rd^ and beyond		
Treatment Type	.98	.60
Chemotherapy Immunotherapy Targeted therapy		
Disease Type	.63	.83
Adenocarcinoma Squamous Cell Small Cell		
	**t**	**p**
Sex		
Male n=36	5.71 (5.0)	t=.48
Female n=64	6.23 (5.5)	p=.64
Race		
Non-White n=14	5.80 (5.6)	t=.19
White n=86	6.10 (5.3)	p=.85
Married		
Yes n=70	5.41 (4.7)	t= 1.88
Non=30	7.52 (6.2)	p=.06
Antidepressant		
Yes n=18	7.03 (5.7)	t=−.58
No n=80	6.04 (5.2)	p=.56

**Table 3:** Multivariate analysis of Neutrophil to Lymphocyte Ratio (NLR) controlled by age, sex, marital status and depression as measured by the Hospital Anxiety Depression Scale.

	Depression		
Variable	Regression Coefficient	*t* value	*P*
Age	.00 (.05)	.031	.98
Sex	.11 (1.1)	1.099	.27
Married	-.11 (1.1)	-1.025	.31
NLR (log transformed)	.21 (1.4)	1.948	.05
	F 2.981 Adjusted R^2^ .16, p=.004		

NLR: neutrophil to lymphocyte ratio.
